# Altered Desaturation and Elongation of Fatty Acids in Hormone-Sensitive Lipase Null Mice

**DOI:** 10.1371/journal.pone.0021603

**Published:** 2011-06-29

**Authors:** Céline Fernandez, Kai Schuhmann, Ronny Herzog, Barbara Fielding, Keith Frayn, Andrej Shevchenko, Peter James, Cecilia Holm, Kristoffer Ström

**Affiliations:** 1 Department of Experimental Medical Science, Lund University, Lund, Sweden; 2 Max Planck Institute of Molecular Cell Biology and Genetics, Dresden, Germany; 3 Oxford Centre for Diabetes, Endocrinology and Metabolism, University of Oxford, Oxford, United Kingdom; 4 Department of Immunotechnology, Lund University, Lund, Sweden; Governmental Technical Research Centre of Finland, Finland

## Abstract

**Background:**

Hormone-sensitive lipase (HSL) is expressed predominantly in adipose tissue, where it plays an important role in catecholamine-stimulated hydrolysis of stored lipids, thus mobilizing fatty acids. HSL exhibits broad substrate specificity and besides acylglycerides it hydrolyzes cholesteryl esters, retinyl esters and lipoidal esters. Despite its role in fatty acid mobilization, HSL null mice have been shown to be resistant to diet-induced obesity. The aim of this study was to define lipid profiles in plasma, white adipose tissue (WAT) and liver of HSL null mice, in order to better understand the role of this multifunctional enzyme.

**Methodology/Principal Findings:**

This study used global and targeted lipidomics and expression profiling to reveal changed lipid profiles in WAT, liver and plasma as well as altered expression of desaturases and elongases in WAT and liver of HSL null mice on high fat diet. Decreased mRNA levels of stearoyl-CoA desaturase 1 and 2 in WAT were consistent with a lowered ratio of 16∶1n7/16∶0 and 18∶1n9/18∶0 in WAT and plasma. In WAT, increased ratio of 18∶0/16∶0 could be linked to elevated mRNA levels of the Elovl1 elongase.

**Conclusions:**

This study illustrates the importance of HSL for normal lipid metabolism in response to a high fat diet. HSL deficiency greatly influences the expression of elongases and desaturases, resulting in altered lipid profiles in WAT, liver and plasma. Finally, altered proportions of palmitoleate, a recently-suggested lipokine, in tissue and plasma of HSL null mice, could be an important factor mediating and contributing to the changed lipid profile, and possibly also to the decreased insulin sensitivity seen in HSL null mice.

## Introduction

Obesity is a growing health problem globally, predisposing individuals to disorders such as type 2 diabetes, cardiovascular disease and certain cancers. Understanding molecular events of fatty acid metabolism in tissues regulating lipid-fluxes e.g. adipose tissue and liver are of great importance in order to understand and reverse the progression of this disease.

HSL is a key enzyme in fatty acid mobilization in white adipose tissue (WAT). It has a wide tissue distribution and is, besides WAT and brown adipose tissue (BAT), expressed also in steroidogenic tissues, skeletal muscle, macrophages, pancreatic β-cells and liver [1]. Its role in catecholamine-stimulated hydrolysis of stored triacylglyceride (TAG) and, in particular, diacylglyceride has been demonstrated in studies of HSL-null mice [2], [3]. Despite its prominent role in lipolysis, HSL-null mice are not obese and exhibit a remarkable resistance to development of obesity following challenge with a long-term high fat diet (HFD) [4], [5]. The resistance to diet-induced obesity is accompanied by impaired adipogenesis [4], [5] and attainment of brown adipocyte features of WAT [4], suggesting that HSL plays additional, yet unexplored, roles in adipose tissue. HSL exhibits broad substrate specificity and besides acylglycerides it hydrolyzes cholesteryl esters, retinyl esters, lipoidal esters and water-soluble esters. The biological significance of these other activities, however, is poorly understood. We have recently shown that HSL is the major retinyl ester hydrolase of white and brown adipocytes [6]. In its absence retinoid metabolism is perturbed, potentially leading to failure to provide retinoic acid for signaling events that are crucial for adipogenesis and determination of adipocyte fate. Thus, in addition to its key role in energy homeostasis, HSL appears to play an important role in the generation of lipids for transcriptional regulation and various lipid signaling events.

It has been reported that metabolic diseases, such as diabetes and obesity, induce changes in hepatic lipid composition by controlling the function of key transcription factors that in turn affect elongase and desaturase expression [7]. This highlights the importance of fatty acid elongation and desaturation in managing hepatic lipid composition in response to dietary status.

Stearoyl-CoA desaturases (SCDs) are ER proteins that catalyze the delta-9-cis desaturation of saturated fatty acids, preferentially 16∶0 and 18∶0. At present time there are four SCD isoforms described in mouse [8]. Whereas expression of SCD3 and 4 is restricted mainly to skin and heart, respectively, a wider tissue distribution is seen for SCD1 and 2. Fatty acid analyses have shown that while mouse SCD3 uses only 16∶0 as a substrate [9] the other isoforms desature both 18∶0 and 16∶0. This, together with tissue distribution, point towards distinct physiological roles of the different SCD. Both SCD1 and 2 are expressed in adipocytes in a differentiation-dependent pattern, where a massive increase of SCD1 and a moderate increase of SCD2 can be seen after differentiation of 3T3-L1 preadipocytes to adipocytes [10], [11]. Also, SCD2 mRNA expression is uniquely increased (44-fold) in adipocytes from mice fed a HFD, with only slight regulation of SCD1 expression [11]. Further, it was recently reported that SCD2, but not SCD1, is required for adipogenesis and maintenance of the adipocyte-specific gene expression in 3T3-L1 adipocytes. SCD1 deficient mice are resistant to HFD-induced obesity and are protected against liver steatosis [12]. It has been suggested that desaturation of saturated fats by SCD1 is an essential step mediating their induction of lipogenesis [13].

Elongase enzymes add two carbon units to the carboxyl end of a fatty acid using malonyl-CoA and fatty acyl-CoA as substrates. Seven distinct fatty acid elongase (Elovl) subtypes are present in the mouse, rat and human genomes (www.ensembl.org), with Elovl1, 3, 6 and 7 preferring saturated and monounsaturated fatty acids and Elovl2, 4 and 5 being selective for polyunsaturated fatty acids [14]. Moreover, Elovls display tissue specificity where Elovl1 appears to be expressed ubiquitously [7], [14]. Elovl3 is expressed mainly in the skin but is also detected in WAT and BAT. Elovl6 is expressed at low levels in several tissues including liver. Finally, Elovl5 is the most abundant elongase in many tissues.

Under normal circumstances, there appears to be a tightly coordinated regulation between glycolysis, de novo lipogenesis, elongation and desaturation, where excess glucose, channeled to de novo lipogenesis is subsequently elongated and desaturated yielding 18∶1, a fatty acid readily stored in TAG and accumulated in livers of obese mice [7].

The aim of the present study was to investigate changes in lipid metabolism of HSL null mice on a HFD, to better understand the role of HSL for these processes. To this end, we performed a targeted lipid analysis of TAG in WAT, liver and plasma and of non-esterified fatty acids (NEFA) in plasma using gas chromatography, as well as a global lipid analysis of plasma using shotgun lipidomics. In addition, we used real-time quantitative PCR technique to measure the gene expression of enzymes involved in desaturation and elongation of fatty acids in WAT, BAT and liver

This study illustrates the importance of HSL for normal lipid metabolism in response to a HFD. HSL deficiency greatly influences the expression of elongases and desaturases, resulting in altered lipid profiles in WAT, liver and plasma. Finally, altered levels of palmitoleate, a recently suggested lipokine, in tissue and plasma of HSL null mice, could be an important factor mediating and contributing to the changed lipid profile, and possibly also to the decreased insulin sensitivity seen in HSL null mice.

## Materials and Methods

### Animal experiments

The study was reviewed and approved by the Ethical Committee in Malmö/Lund, Lund, Sweden (license no. M202-08) and is in accordance with the Council of Europe Convention (ETS 123). HSL null mice were generated by targeted disruption of the HSL gene in SV129-derived embryonic stem cells as described elsewhere [15]. Animals used were from the same embryonic stem cell colony and animals in the different groups were littermates and had a mixed genetic background from the inbred strains C57BL/6J and SV129 [2]. Both male and female mice were studied. The animals were maintained in a temperature-controlled room (22oC) on a 12-h light-dark cycle. The diet studies were initiated by feeding mating mice a high fat diet (HFD) (58% energy from fat) (Research Diets; products no D12309 and D05021605). The mice were maintained on a HFD, provided *ad libitum,* until the age of 5 months, after which the mice were killed and tissues dissected. Collected tissues were rapidly dissected, and tissues and plasma were snap frozen and stored in liquid nitrogen before analyses. Blood samples were drawn by retro-orbital puncture. Tissues and plasma were collected after an overnight fast (12–6 h). Gene expression was analyzed using real-time quantitative PCR technique, on RNA extracted from isolated periovarial white adipocytes, BAT and liver from female mice after an overnight fast (12–16 h).

### Preparation of isolated white adipocytes

Adipocytes from periovarial WAT were isolated after incubation in Krebs-Ringers solution (pH 7.4) supplemented with 3.5% BSA, 2 mM glucose, 200 nM adenosine and collagenase (1 mg/ml; Sigma) in a shaking incubator at 37°C for ∼60 min according to a modification of the Rodbell method [16]. The digested tissue was filtered and the isolated cells were washed twice in Krebs-Ringer buffer (pH 7.4) with 1% BSA, 2 mM glucose and 200 nM adenosine by allowing the isolated adipocytes to float to the surface and then aspirate the underlying buffer.

### Tissue homogenate preparation

Periovarial WAT was homogenized in 0.25 M sucrose, 1 mM EDTA pH 7.0, 1 mM dithiothreitol, 20 µg/ml leupeptin, 10 µg/ml antipain, 1 µg/ml pepstatin A using a glass/teflon homogenizer (10–20 strokes), followed by a centrifugation at 10 000 g for 25 min at 4°C. The infranatant fraction was used for western blot detection of FAS. The pellet fraction was solubilized in homogenization buffer containing 5% SDS for 30 min at 50°C, and was used for western blot detection of SREBP1. Protein determination was performed using the BCA assay (Pierce).

### RNA preparation and Real-time quantitative PCR (rtPCR)

Total RNA was isolated using RNeasy Lipid Tissue Mini Kit (Qiagen) according to the manufacturer's recommendations. Total RNA (1 µg) was treated with DNase I (DNase I amplification grade, Invitrogen) and then reversely transcribed using random hexamers (Amersham Biosciences) and SuperScript™II RNaseH reverse transcriptase (Invitrogen Life Technologies) according to the manufacturer's recommendations. The cDNA was used in quantitative PCR reactions using Taqman chemistry (assays-on-demand, Applied Biosystems) with an ABI 7900 system (Applied Biosystems). The assay Ids of the Taqman probes are listed below. Relative abundance of mRNA was calculated with normalization by geometric averaging of two internal control genes (TATA box binding protein (TBP) and ribosomal protein S29 (RPS29) for adipocytes and using three internal control genes (glyceraldehyde 3-phosphate dehydrogenase (GAPDH), hypoxanthine-guanine phosphoribosyl transferase (HPRT) 1 and RPS29) for liver samples [17].

### Primers used in real-time quantitative PCR experiments

List of assays using Taqman probes:

**SCD1** (Assays-on-demand, Mm00772290_m1)

**SCD2** (Assays-on-demand, Mm00485951_g1)

**Elovl1** (Assays-on-demand, Mm00517077_m1 and Mm01188316_g1 (liver))

**Elovl3** (Assays-on-demand, Mm00468164_m1 and Mm01194164_m1 (liver))

**Elovl5** (Assays-on-demand, Mm00506717_m1)

**Elovl6** (Assays-on-demand, Mm00851223_s1 and Mm04209852_g1 (liver))

**TBP** (Assays-on-demand, Mm00446973_m1)

**RPS29** (Assays-on-demand, Mm02342448_gH)

**GAPDH** (Assays-on-demand, Mm99999915_g1)

**HPRT1** (Assays-on-demand, Mm01545399_m1)

### Western blot analysis

Periovarial WAT was homogenized and 25–50 µg of total protein were resolved by SDS-PAGE and electroblotted onto nitrocellulose membranes (HyBond-c extra, Amersham Pharmacia Biotech). Primary antibodies used were SREBP1, fatty acid synthase (FAS) (Santa Cruz Biotechnology), β-actin and α-tubulin (Sigma-Aldrich). Actin and tubulin were used as loading controls. Secondary antibodies were horseradish peroxidase-conjugated anti-rabbit IgG (SREBP1 and FAS) and anti-mouse IgG (α-tubulin and β-Actin) (Amersham). Western blot analysis was performed using a chemiluminescence system (Luminol) and detection was made using a CCD-camera (LAS 1000, Fuji). Relative protein levels were calculated after normalization to the loading control.

### Analysis of fatty acid composition of plasma NEFA, plasma TAG, WAT TAG and liver TAG

Lipids from plasma or homogenized WAT and liver were extracted in 2∶1 chloroform: methanol (v/v) [18]. The solvent layer was evaporated to dryness and lipid classes were separated by solid-phase extraction [19] and fatty acid methyl esters (FAME) prepared using acidic methanol before analyzing the samples by gas chromatography [20]. Fatty acids were identified using specific fatty acid methyl esters and verified on each run using a standard containing methyl esters of fatty acids ranging from chain length 6 to 24 (Sigma-Aldrich Company Ltd, Poole, Dorset, UK). In addition, a methyl ester standard of known fatty acid composition was run with every set of samples (AOCS std#6, Thames Restek UK Ltd, Saunderton, Bucks, UK) to check correct identification of sample peaks and performance of the instrument. The mean and between-run precision (CV (%), n = 30) was as follows 16∶0 29.7 (0.62), 16∶1n7 30 (0.96), 18∶0 14.0 (0.79) and 18∶1n9 41.7 (0.52). A quality control sample was also run with each set of samples. This was a mixture of fatty acids (Sigma-Aldrich Company Ltd) and TG (MaxEPA fish oil, Seven Seas Ltd, Marfleet, Hull UK) that was extracted, separated and methylated alongside each set of samples. The mean and between-assay precision (CV (%), n = 33) was as follows for TG 16∶0 25 (4.0), 16∶1n7 12 (5.7), 18∶0 5.9 (6.2) and 18∶1n9 16.4 (4.2). The mean and within-assay precision (CV (%), n = 22) was calculated on replicate measurements of human adipose tissue as follows 16∶0 22.5 (1.6), 16∶1n7 5.3 (1.4), 18∶0 4.4 (2.3) and 18∶1n9 46.0 (1.03). On a GC, the peak area is proportional to the weight of the fatty acid, and using the peak areas, results are expressed as weight % (g/100 g total fatty acids).

### Shotgun screening of plasma lipidome

Plasma lipid extraction was performed as previously described [21]. In brief, 5 µL of plasma were placed into an eppendorf tube and 350 µL of a standard mixture consisting of the following lipids was added: cholesteryl heptadecanoate (CE 17∶0), 3.6 nmol; heptadecanoyl sphingomyelin (SM 17∶0), 0.9 nmol; 1,2-di-O-hexadecyl-*sn*-glycero-3-phosphocholine (PC-O12∶0/-O12∶0), 3.2 nmol; 1,2-di-O-phytanyl-*sn*-glycero-3-phosphoethanolamine (PE-O16∶0/-O16∶0), 1.3 nmol; 1-lauroyl-2-hydroxy-*sn*-glycero-3-phosphocholine (LPC 12∶0), 3.0 nmol (Avanti Polar Lipids Inc, Alabaster, AL), trilaurin (TAG 12∶0), 2.9 nmol and dilaurin (DAG 12∶0), 0.4 nmol (Larodan, Fine Chemicals, Malmö, Sweden). Then 350 µL of methyl-tert-butylether (MTBE) (Sigma-Aldrich) were added and the samples were shaken at 4°C for 1 h. Afterwards 150 µL of water (LC-MS grade) (Merck, Darmstadt, Germany) were added, followed by shaking at 4°C for 10 min and centrifugation for 5 min at 4,000 rpm on a minispin centrifuge (Eppendorf, Hamburg, Germany). The upper phase was transferred into a glass vial and stored at −20°C until analysis. Mass spectrometric analysis was performed on a LTQ Orbitrap (Thermo Fisher Scientific, Waltham, MA) coupled to a TriVersa NanoMate robotic nanoflow ion source (Advion BioSciences, Ithaca, NY) as previously described [22]. Samples were analyzed in duplicate. Lipids were identified and quantified using the LipidXplorer software [22] and lipid species of the following lipid classes were recognized and quantified: TAG, diacylglycerides (DAG), cholesteryl esters (Chol-FA), sphingomyelins (SM), phosphatidylcholines (PC), phosphatidylcholine ethers (PC-O), lysophophatidylcholines (LPC), phosphatidylethanolamines (PE) and phosphatidylethanolamine ethers (PE-O). Identification of the different lipid species was based on MS survey scans acquired in positive ion mode in the Orbitrap analyzer at a target mass resolution of 100,000 using a mass accuracy of better than 4 ppm and an occupancy threshold of at least 2,000 counts per peak area. Only peaks with an intensity signal at least 5 times above the blank were further considered. Species were quantified by normalizing the intensities of their peaks to the intensity of the peaks of internal standards spiked into the sample prior to lipid extraction.

### Glucose and Insulin Tolerance Test

Intravenous glucose tolerance test (IGTT) and insulin tolerance test (ITT) were performed on female mice on 18 weeks HFD (ITT) or 24 weeks HFD (IGTT).

For the IGTT, D-glucose (1 g/kg) was injected into the tail vein of anesthetized mice (midazolam (0.4 mg/mouse; Dormicum®; Hoffmann-La Roche) and a combination of fluanison (0.9 mg/mouse) and fentanyl (0.02 mg/mouse; Hypnorm®; Janssen, Beerse, Belgium)) after 6 h daytime food withdrawal. In the ITT, 0.75 milliunits/g human insulin (Actrapid; Novo Nordisk, Bagsvaerd, Denmark) was given intraperitoneally to non-fasted anesthetized mice. Plasma glucose and insulin levels were determined in retro-orbital blood samples collected at the time points indicated in the figures.

### Statistics

Data are expressed as mean ± SEM unless otherwise stated. Statistical analysis was made using a non-parametric Mann-Whitney U test. Data were considered significant if p<0.05. p<0.05 = *, p<0.01 = **, p<0.001 = ***. Univariate statistical analyses were performed with the Graph Pad Prism software version 4 (GraphPad, San Diego, CA). Multivariate statistical methods were also applied to the lipidomics data set using SIMCA-P+12.0 (Umetrics, Umeå, Sweden). An Orthogonal Partial Least Squares-Discriminant Analysis (OPLS-DA) was carried out to the pareto-scaled data to identify the discriminant lipid species between the two studied groups.

## Results

### Decreased desaturation of fatty acids in adipose tissue, plasma and liver of HSL null mice on HFD

**Figure 1:**
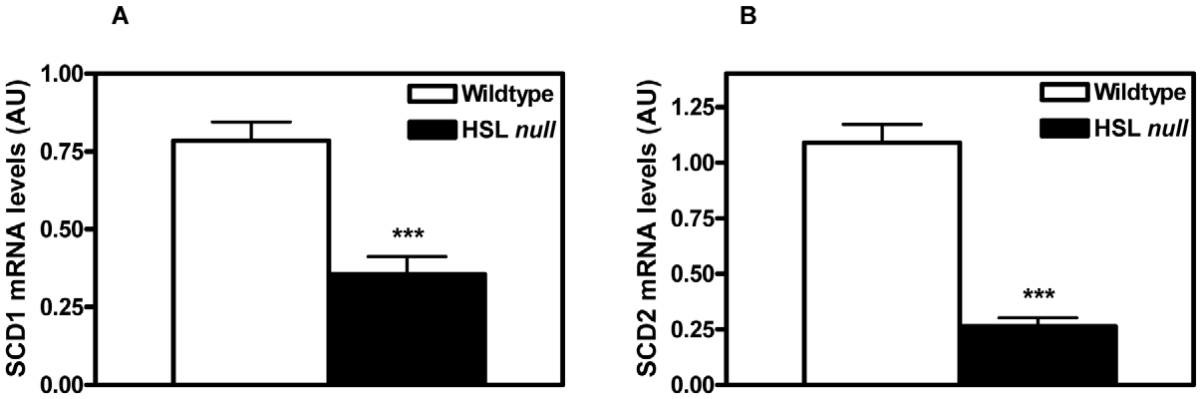
Decreased expression of 9-desaturases in white adipocytes from HSL null mice. Level of mRNA of SCD1 (A) and SCD2 (B) analyzed with real-time quantitative PCR in isolated adipocytes from periovarial WAT from 5 months old, HFD-fed wildtype and HSL null mice after an overnight fast (n = 11). Values given are mean ± SEM, ***p<0.001, analyzed with Mann-Whitney U tests.

Confirming previous results of a dramatic decrease in SCD1 at the protein level in WAT from HSL null mice [4], we here show decreased SCD1 mRNA levels (0.45, p<0.001) in isolated white adipocytes of HSL null mice (**Figure 1A**). Also, the mRNA level of SCD2 was dramatically decreased (0.24, p<0.001) in isolated white adipocytes of HFD-fed HSL null mice compared to wildtype littermates (**Figure 1B**). This is in contrast to the liver, where a significant increase in the expression level of SCD1 [23] and SCD2 (2.98, p<0.01) (**Table 1**) was seen in HSL null mice in the fasted state. In BAT, mRNA levels of SCD1 and 2 were unchanged between the two genotypes (**Table 1**).

**Table 1:** Gene expression analysis in liver and BAT of wildtype and HSL null mice.

	Liver			BAT		
Gene	Wildtype	HSL null	P-value	Wildtype	HSL null	P-value
SCD1	See [23]			0.89±0.14	0.84±0.20	1.00
SCD2	0.35±0.01	1.03±0.20**	0.008	0.94±0.13	0.89±0.10	0.84
Elovl1	0.84±0.03	1.04±0.05*	0.016	0.37±0.01	0.76±0.09**	0.008
Elovl3	0.35±0.06	0.54±0.04	0.057	0.79±0.13	0.20±0.07*	0.016
Elovl5	0.72±0.10	0,53±0.11	0.13	0.78±0.05	0.77±0.07	1.00
Elovl6	0.88±0.16	0.82±0.09	0.73	0.91±0.13	0.83±0.17	0.69

Data were generated using Real-time quantitative PCR on female mice after HFD followed by an overnight fast (12 h). Values given are arbitrary means ± SEM, n = 5. Relative mRNA quantities were normalized by geometric averaging of three internal control genes (RPS29, HPRT1 and GAPDH) for the liver and two internal control genes (RPS29 and TBP) for BAT. Differences between the two genotypes were analyzed using a non-parametric Mann-Whitney U-test. Data were considered significant if p<0.05.

*p<0.05,

**p<0.01.

**Figure 2:**
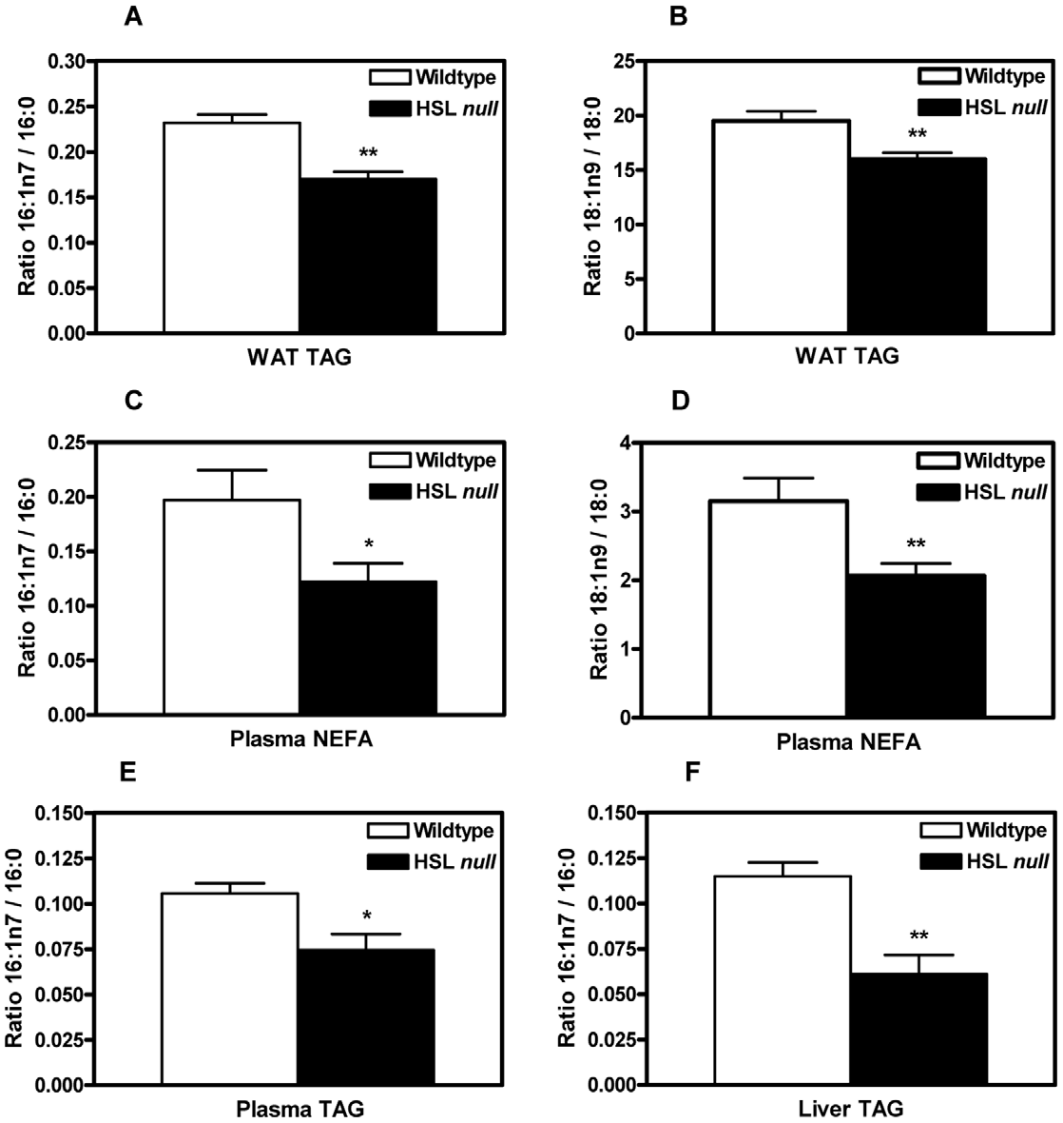
Decreased desaturation of fatty acids in WAT, plasma and liver from HSL null mice. Ratios of 16∶1n7/16∶0 (A) and 18∶1n9/18∶0 (B) fatty acids in epididymal WAT TAG from 5 months HFD-fed HSL null mice (n = 6–12). Ratios of 16∶1n7/16∶0 (C) and 18∶1n9/18∶0 (D) fatty acids in plasma NEFA (n = 6–10) and 16∶1n7/16∶0 (E) fatty acids in plasma TAG (n = 6–11) in 5 months old HFD-fed male wildtype and HSL null mice. (F) Ratios of 16∶1n7/16∶0 fatty acids in liver TAG from female mice (n = 6–7). Tissues and plasma were collected from mice after an overnight fast. Values given are mean ± SEM, *p<0.05, **p<0.01, analyzed with Mann-Whitney U tests.

Fatty acid ratios in tissue or plasma lipid classes are frequently used to mirror enzyme activity of e.g. desaturases and elongases. Moreover, fasting plasma NEFA and TAG may reflect enzyme activities in adipose tissue and liver, respectively [24]. Accordingly, decreased expression of Δ9-desaturases in white adipocytes was reflected in decreased ratios of 16∶1n7/16∶0 (0.73, p<0.01) and 18∶1n9/18∶0 (0.82, p<0.01) fatty acids in WAT TAG from HFD-fed HSL null mice (**Figure 2A,B**). In plasma, decreased desaturase activity in WAT resulted in lower ratios of 16∶1n7/16∶0 (0.66, p<0.05) and 18∶1n9/18∶0 (0.66, p<0.01) NEFA (**Figure 2C,D**). The lowered ratio of 16∶1n7/16∶0 fatty acids (0.70, p<0.05) seen in plasma TAG (**Figure 2E**) and the markedly lower ratio of 16∶1n7/16∶0 (0.53, p<0.01) in the liver (**Figure 2F**), suggest a decreased desaturase activity in the liver of HSL null mice, whereas SCD1 mRNA is in fact increased as above. Such a discrepancy between SCD1 expression and 16∶1n7/16∶0 ratio has been reported previously [25], and could be the result of altered expression and activity of other enzymes involved in lipid metabolism, e.g. elongases. Decreased proportions of 16∶1n7 palmitoleic acid were seen in WAT TAG (0.74, p<0.01), plasma NEFA (0.70, p<0.05) and plasma TAG (0.63, p<0.05) of HSL null mice on a HFD (**Table 2**). Also, proportions of 16∶0, 18∶0 and 18∶1n9 in WAT TAG, plasma NEFA and plasma TAG in HSL null mice compared to wildtype are listed in **Table 2**.

**Table 2:** Fatty acid profile in WAT and plasma of wildtype and HSL null mice.

Lipid species	Genotype	16∶0	16∶1n7	18∶0	18∶1n9
WAT TAG	Wildtype	16.28±0.46	3.77±0.19	3.03±0.10	58.76±0.92
	HSL null	16.49±0.39	2.78±0.12**	3.74±0.13**	59.02±0.46
		p = 0.89	p = 0.002	p = 0.002	p = 0.89
Plasma NEFA	Wildtype	21.76±0.91	4.22±0.48	12.42±0.84	38.58±0.87
	HSL null	24.73±0.75*	2.95±0.26*	17.06±0.73**	34.76±1.02**
		p = 0.016	p = 0.029	p = 0.003	p = 0.008
Plasma TAG	Wildtype	20.11±0.51	2.12±0.09	4.43±0.68	42.53±2.57
	HSL null	17.60±0.52*	1.33±0.18*	4.24±0.21	37.10±1.00
		p = 0.010	p = 0.018	p = 0.74	p = 0.068

Values given are means ± SEM, in g/100 g of total fatty acids. Data were generated on male mice after HFD followed by an overnight fast (12 h) (n = 6–12). Differences between the two genotypes were analyzed using a non-parametric Mann-Whitney U-test. Data were considered significant if p<0.05.

*p<0.05,

**p<0.01.

### Altered expression of elongases in adipocytes HSL null mice on HFD

**Figure 3:**
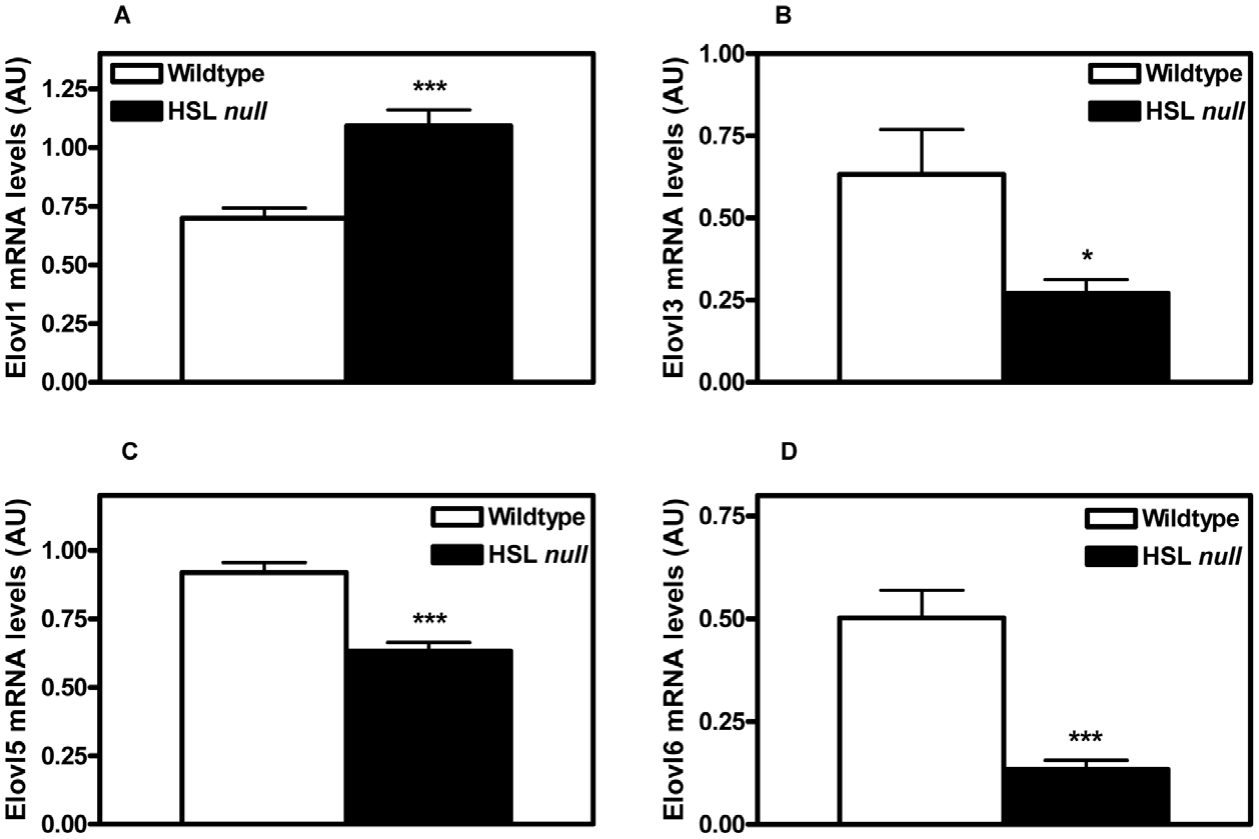
Altered expression of elongases in white adipocytes from HSL null mice. Level of mRNA of Elovl1 (**A**), Elovl3 (**B**), Elovl5 (**C**) and Elovl6 (**D**) analyzed with real-time quantitative PCR in isolated adipocytes from periovarial WAT from 5 months old, overnight fasted wildtype and HSL null mice fed a HFD (n  =  11). Values given are mean ± SEM, *p<0.05, ***p<0.001, analyzed with Mann-Whitney U tests.

Elovl1 is commonly regarded as a housekeeping gene in many tissues and is often seen expressed at steady levels [14]. When investigating the expression levels of different elongases in isolated adipocytes from WAT, a striking result was a significant increase in the mRNA levels of Elovl1 (1.56, p<0.001) in adipocytes from HSL null mice fed a HFD (**Figure 3A**). In contrast, decreased expression levels of Elovl3 (0.43, p<0.05), Elovl5 (0.69, p<0.001) and Elovl6 (0.27, p<0.001) were seen in adipocytes from HSL null mice on a HFD (**Figure 3B–D**). In the liver, increased levels of Elovl1 (1.24, p<0.05) and 3 (1.85, p = 0.057) were seen in HSL null mice on HFD, whereas the levels of Elovl 5 and 6 were not significantly changed between the two genotypes (**Table 1**). Similar to WAT, increased levels of Elovl1 (2.05, p<0.01) and decreased levels of Elovl3 (0.26, p<0.05) were also observed in BAT of HSL null mice fed HFD (**Table 1**). However, in BAT, levels of Elovl5 and 6 were unchanged between the two genotypes (**Table 1**).

### Increased elongation of fatty acids in WAT, plasma and liver from HSL null mice on HFD

**Figure 4:**
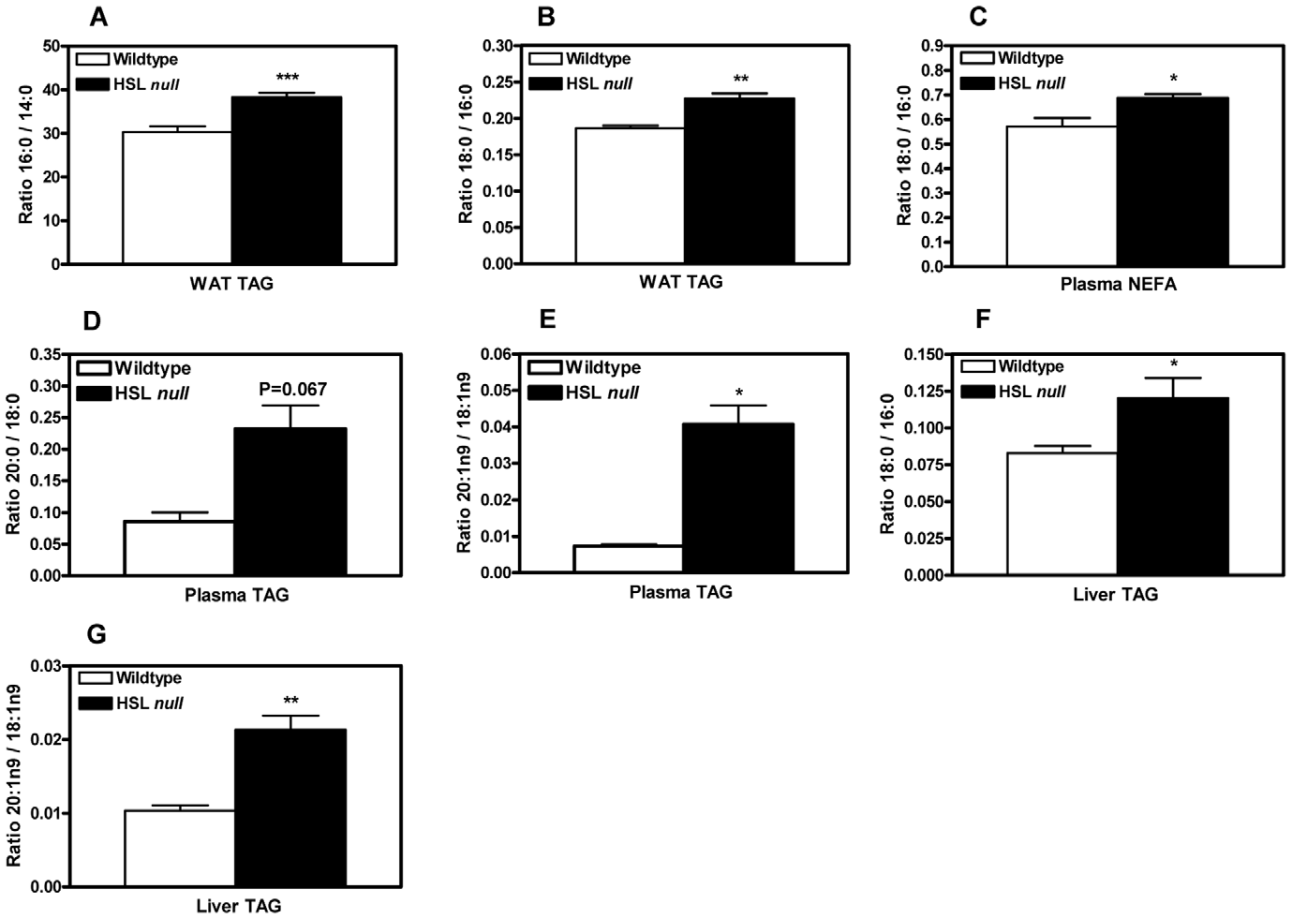
Increased elongation of fatty acids in WAT, plasma and liver of HSL null mice. Ratios of 16∶0/14∶0 (A) and 18∶0/16∶0 (B) fatty acids in epididymal WAT TAG from 5 months old HFD-fed HSL null mice (n = 6–12). Ratios of 18∶0/16∶0 (C) plasma NEFA (n = 6–10), and 20∶0/18∶0 (D) and 20∶1n9/18∶1n9 (E) fatty acids in plasma TAG (n = 3–7) of 5 months old male wildtype and HSL null mice fed HFD. Ratios of 18∶0/16∶0 (F) and 20∶1n9/18∶1n9 (G) fatty acids in liver TAG (n = 5–7) of 5 months old female wildtype and HSL null mice fed HFD. Tissues and plasma were collected from mice after an overnight fast. Values given are mean ± SEM, *p<0.05, **p<0.01, ***p<0.001, analyzed with Mann-Whitney U tests.

Elevated ratios of 16∶0/14∶0 (1.26, p<0.001) and 18∶0/16∶0 (1.22, p<0.01) fatty acids were seen in WAT TAG from HFD-fed HSL null mice (**Figure 4A,B**). Similarly, in plasma from HFD-fed HSL null mice, a higher ratio of both 18∶0/16∶0 (1.21, p<0.05) NEFA (**Figure 4C**), and 20∶0/18∶0 (2.70, p = 0.067) and 20∶1n9/18∶1n9 (5.57, p<0.05) fatty acids in plasma TAG (**Figure 4D,E**) was observed**.** In the liver TAG from HFD-fed HSL null mice, elevated ratios of 18∶0/16∶0 (1.45, p<0.05) and 20∶1n9/18∶1n9 (2.06, p<0.01) fatty acids were seen (**Figure 4F,G**).

### Dramatic reduction of plasma TAG molecular species in HSL null mice on HFD

**Figure 5:**
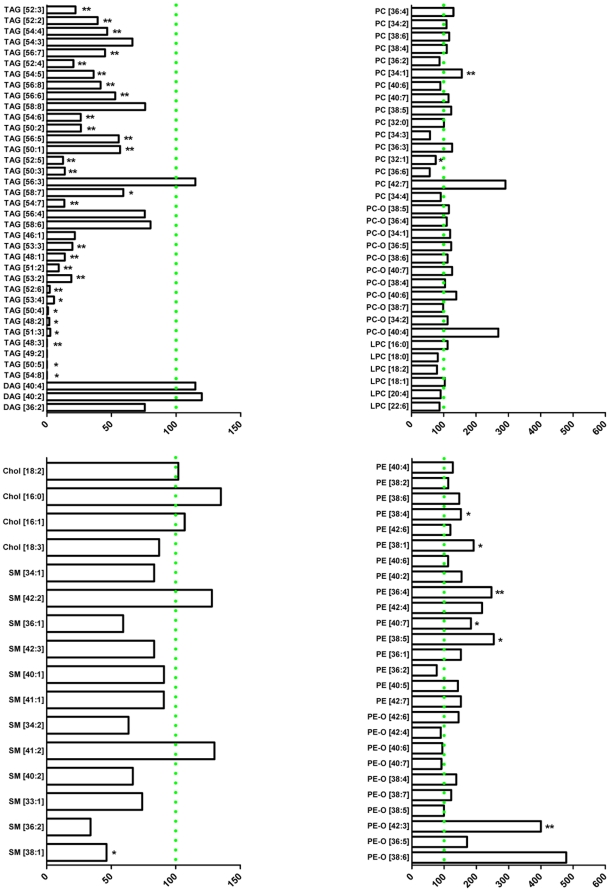
Plasma lipidome analysis by top-down shotgun mass spectrometry of HFD-fed HSL null and wildtype mice. Data are expressed as % change of a lipid molecular species in HSL null vs. wildtype female mice with values below 100% fold change threshold indicating decreased amount and values above the 100% fold change threshold suggesting increased amount in HSL null vs. wildtype. Different lipid species are ranked within each class in descending order according to their absolute abundance in HSL null mice. Data is presented as median value (n = 4–6), *p<0.05, **p<0.01, analyzed with Mann-Whitney U test. No correction for multiple testing was done and by chance only 5.7 (113*0.05) lipid species would be expected to be differentially expressed between the two genotypes.

A total of 113 lipid species belonging to 9 different lipid classes were identified and quantified in mouse plasma by shotgun lipidomics, of which 35 TAGs, 3 DAGs, 4 Chol-FAs, 12 SMs, 16 PCs, 11 PC-Os, 6 LPCs, 16 PEs and 10 PE-Os (**Figure 5**). Most of the changes in the lipid profile of HSL null mice compared to wildtype occurred within the TAG lipid class where the plasma content of 28 out of the 35 analyzed TAG species was diminished (p<0.05 and p<0.01) and most of the regulated TAG species were decreased by more than 50%. Other affected lipid species included SM 38∶1, the levels of which were halved in HSL null mice compared to wildtype (p<0.05). PE species tended to be elevated in HSL null mice compared to control, e.g. PE36∶4 was increased 2.5 fold, and statistical significance was reached for 5 PE species out of the 16 measured (p<0.05 and p<0.01).

**Figure 6:**
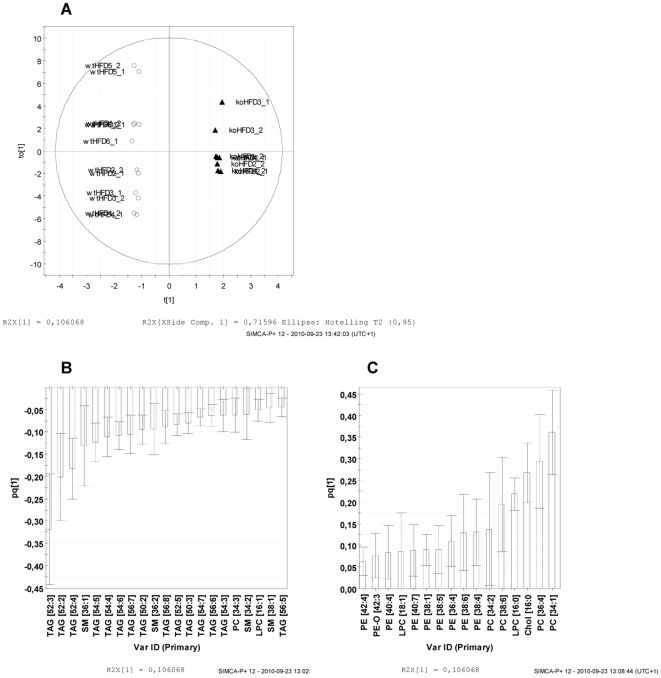
Plasma lipidomics reveals differences between HSL null mice and wildtype mice following a HFD. Orthogonal Partial Least Squares-Discriminant Analysis (OPLS-DA) of plasma lipidomics data was applied to differentiate the HSL null mice on HFD from the control mice with 113 identified lipid species included in the analysis as variables. (**A**) Score plot illustrating clustering of the samples according to the genotype, with each data point on the plot representing the individual plasma lipid profile of an animal. (**B**,**C**) Loading column plots to visualize the class-separating lipid species with a T-bar corresponding to the 95% confidence interval. Lipid species decreased (**B**) and increased (**C**) in plasma of female HSL null mice on HFD vs. wildtype littermates.

Apart from the univariate data analysis, OPLS-DA of the plasma lipidomics data was conducted to establish the discriminant lipid species between the two groups with the 113 identified lipid species included in the analysis as variables. A clear separation between HSL null mice on HFD and wildype littermates was achieved (**Figure 6A**). Thirty-seven lipid species contributed the most to the separation between the two genotypes (**Figure 6B,C**). Several TAG species as well as SM 38∶1 were found to be less abundant (**Figure 6B**), while several PE species were found to be elevated (**Figure 6C**) in HSL null mice on HFD vs. wildype littermates, supporting the results from the univariate data analysis.

### Decreased protein levels of lipogenic genes in WAT of HSL null mice fed a HFD

**Figure 7:**
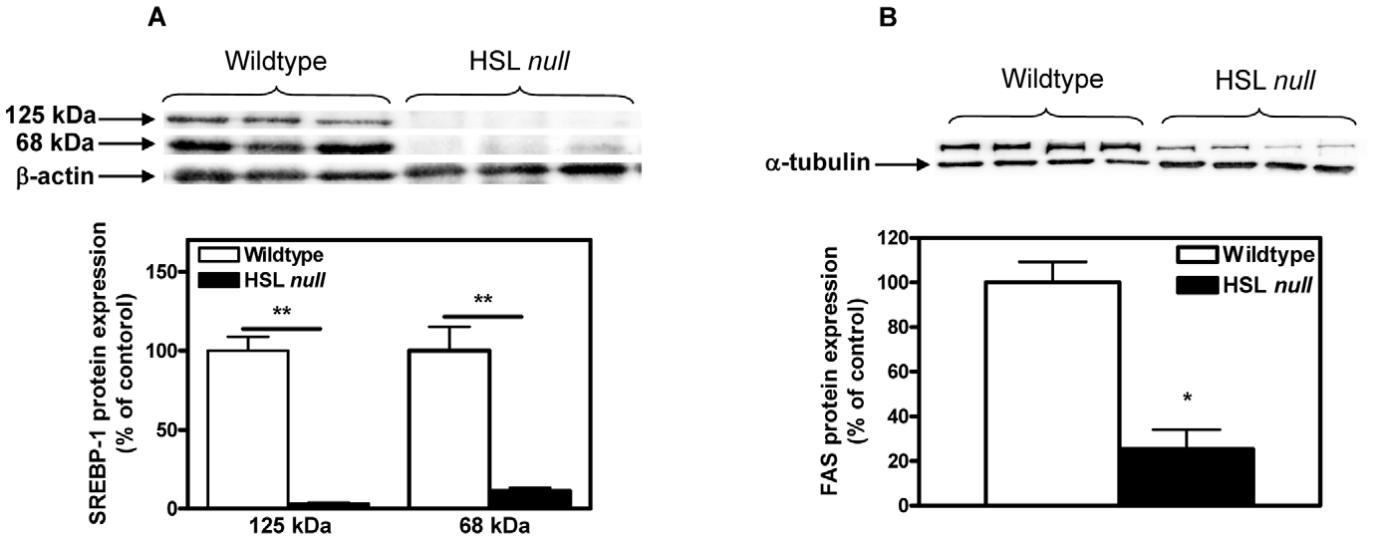
Decreased levels of lipogenic enzymes in WAT of HSL null mice fed a HFD. Protein levels of SREBP1 (A) and FAS (B), analyzed with western blot in WAT from 5 months old female wildtype and HSL null mice fed HFD (n = 4–7). Values given are mean ± SEM, *p<0.05, **p<0.01, analyzed with Mann-Whitney U tests.

To further characterize the metabolic state and the capacity of WAT to generate endogenous fatty acids, protein levels of key enzymes in de novo lipogenesis were analyzed in WAT of HSL null mice fed a HFD. Decreased mRNA levels of SREBP1c and FAS has previously been demonstrated in WAT [5] and in isolated adipocytes of HSL null mice on a HFD [6]. We here show dramatically decreased protein levels of both the inactive ER-bound precursor (125 kDa) (0.03, p<0.01) and the active, nuclear located DNA-binding isoform (68 kDa) (0.11, p<0.01) of SREBP1c (**Figure 7A**) and of FAS (0.25, p<0.05) (**Figure 7B**) in WAT from HSL null mice fed a HFD.

### HSL null mice on a HFD show signs of impaired insulin sensitivity

**Figure 8:**
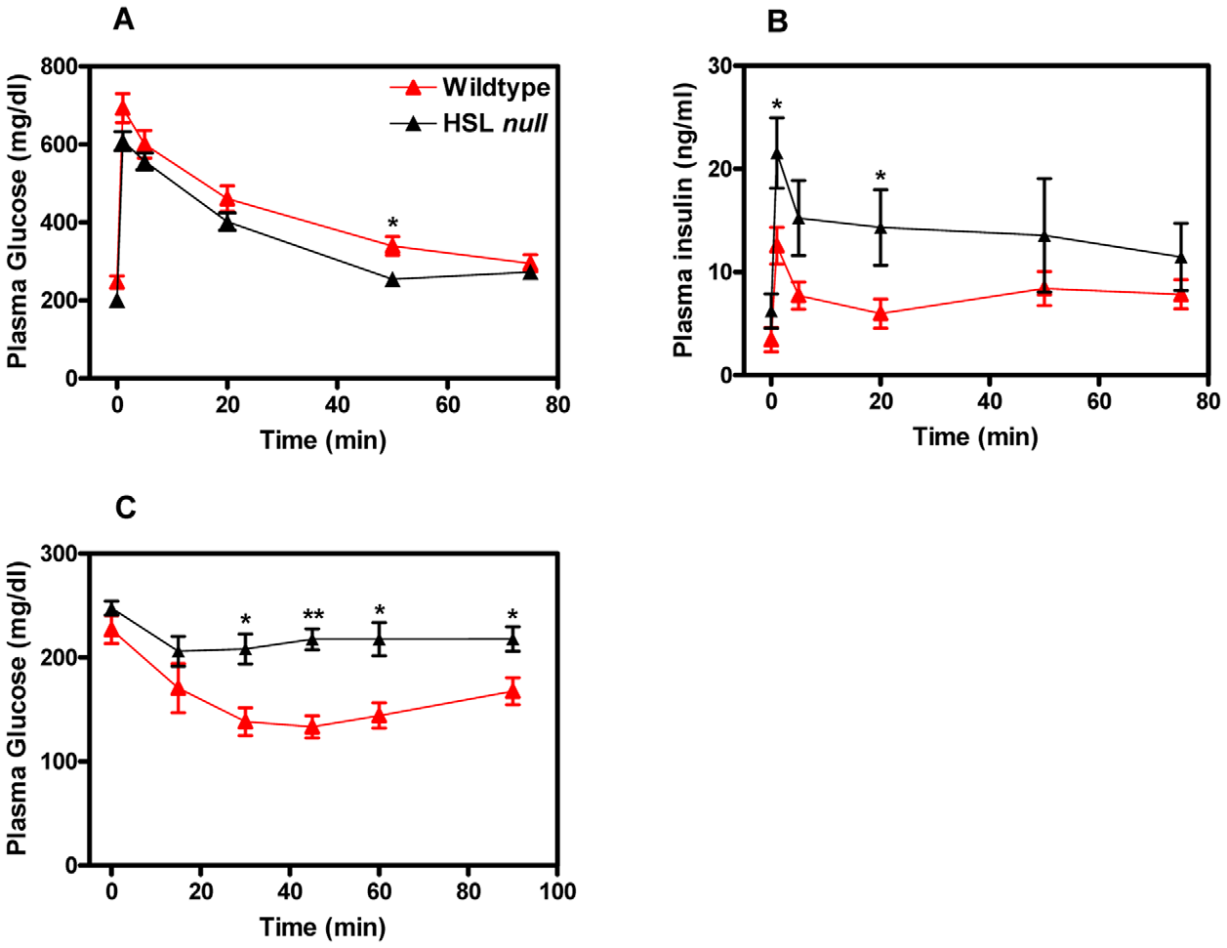
HSL null mice on a HFD show signs of impaired insulin sensitivity. Plasma levels of glucose (A) and insulin (B) after an intravenous glucose tolerance test (IGTT) performed on fasted (6 h) female mice after 24 weeks of HFD (n = 5–6). (C) Plasma insulin levels after an insulin tolerance test (ITT) performed on non-fasted female mice after 18 weeks of HFD (n = 6). Values given are mean ± SEM, *p<0.05, **p<0.01, analyzed with Mann-Whitney U tests.

HSL null mice generated in our lab show signs of impaired insulin sensitivity when fed a normal chow diet [2]. To investigate the impact of diet on the sensitivity to insulin, glucose and insulin tolerance tests were performed in mice on HFD. The intravenous glucose tolerance test (IGTT) showed that whereas plasma levels of glucose (**Figure 8A**) were similar between the two genotypes, levels of insulin (**Figure 8B**) were significantly elevated in HSL null mice (p<0.05). Also, increased glucose levels in HSL null mice after an insulin tolerance test (ITT) (p<0.05 and p<0.01) (**Figure 8C**), suggest impaired insulin sensitivity in these mice.

## Discussion

In this study we report a markedly decreased expression of SCD1 and 2 in WAT of HSL null mice on a HFD. Decreased activity of SCDs in WAT is reflected in lowered desaturation ratios of palmitate and stearate in WAT TAG and plasma NEFA. In the liver, decreased desaturation of palmitate is seen despite increased expression of SCD1. Also, elevated mRNA expression of Elovl1 in WAT from HSL null mice on HFD, possibly underlie the increased elongation of palmitate observed in WAT TAG and plasma NEFA.

SCDs are critical regulators of energy metabolism and catalyze the synthesis of monounsaturated fats, the most important ones being 16∶1n7 and 18∶1n9. SCD1 catalyzes the conversion of 18∶0 to 18∶1n9, a major substrate for triglyceride synthesis, and is proposed to be required for the development of diet-induced obesity and insulin resistance [8]. Mice with a global deletion of SCD1 are resistant to HFD and genetically induced obesity and are protected against liver steatosis in a number of mouse models [12], [26]. Reduced adiposity is associated with increased insulin sensitivity [12]. It has recently been suggested that hepatic SCD1 expression, via the generation of oleate, is required for carbohydrate-induced adiposity, whereas SCD1 inhibition in extrahepatic tissue is required to protect mice from HFD-induced obesity and insulin resistance [27]. Mice with liver specific SCD1 deletion show decreased lipogenesis and lower levels of nuclear SREBP1. In the HSL null mice, a clear connection can be seen with tissue SCD1 expression and TAG incorporation. In WAT, where SCD1 levels are decreased (**Figure 1A**), TAG incorporation is significantly lower [5]. The opposite is seen for the liver having increased expression of SCD1 [23] and increased incorporation of TAG [5]. Similar to SCD1 deficient mice [8] HSL null mice have decreased desaturation index (16∶1n7/16∶0 and 18∶1n9/18∶0) in plasma, and also in WAT. In liver of HSL null mice, however, the proportion of 18∶1n9 is increased [23] possibly mediated by increased SCD1 activity. The accumulation of 18∶1 in the liver is a typical feature of liver steatosis [7].

In cultured 3T3-L1 adipocytes, SCD2 is correlated to and required for the induction and maintenance of PPARγ protein levels and for adipogenesis [11]. It is suggested that rather than providing fatty acid signals to PPARγ-dependent pathways, SCD2 could be important for the generation of unsaturated FAs necessary for normal functions of the transcriptional machinery that drives PPARγ expression and also to maintain protein synthesis rates of PPARγ in mature adipocytes. Adipocytes from HSL null mice on a HFD show markedly decreased (4-fold) levels of SCD2 (**Figure 1B**) and lower levels of the master regulators of adipogenesis i.e. PPARγ and C/EBPα [4]. These data are in agreement with SCD2 being in control of PPARγ expression and adipogenesis. It has been speculated that PPARγ plays a role as fatty acid sensor, allowing proper expression of FA metabolizing enzymes and the generation of new adipocytes [11]. Differentiating adipocytes can fully synthesize a PPARγ ligand, since preadipocytes will differentiate and produce a PPARγ ligand in the absence of exogenous FAs [28], [29]. These findings stress the importance of endogenously synthesized FAs for adipogenesis.

16∶1n7 fatty acid has recently been identified as a potential lipokine derived from adipose tissue that contributes to the regulation of global lipid homeostasis [30]. Mice lacking both FABP4 and 5 (FABP^−/−^ mice) are strongly protected from diet-induced obesity, insulin resistance, type-2 diabetes and liver steatosis [31]. The adipose tissue of FABP^−/−^ mice is resistant to diet-induced insulin resistance and the lipid profile of adipose tissue is strikingly enriched in 16∶1n7 fatty acid in all major lipid classes [30]. The suppressive effect of HFD on the level of 16∶1n7 in adipose tissue seen in wildtype mice is lost in FABP^−/−^ mice. Also, levels of palmitoleate in plasma NEFA of FABP^−/−^ mice were increased, illustrating the importance of WAT as a source of circulating free fatty acids. Intriguingly, after a HFD regimen, TAG in WAT from HSL null mice contains a significantly less proportion of 16∶1n7 fatty acids (**Table 2**). This could be a contributing factor to the decreased sensitivity to insulin previously shown in this tissue [2], [5]. Further, circulating levels of 16∶1n7 in plasma NEFA, are significantly decreased, most likely reflecting lipid metabolism in WAT, and is a possible confounding factor to the observed systemic decrease in insulin sensitivity of HSL null mice on HFD (**Figure 8**).

Hepatic SCD1 activity is dramatically suppressed in FABP^−/−^ mice [31]. It has recently been shown that palmitoleate is the main lipid component contributing to the suppression of SCD1 expression in liver cells [30]. In WAT, a marked stimulation of FAS, SCD1 and ELOVL6, three principal enzymes mediating de novo lipogenesis, is seen in FABP^−/−^ mice [30]. This highlights the opposite pattern of expression of SCD1, and other lipogenic genes in liver vs. adipose tissue of FABP^−/−^ mice, likely regulated by 16∶1n7. However, in skeletal muscle of these mice, another main target of insulin, the regulation of SCD1 activity follows that of WAT [31]. Also, concentrations of muscle palmitoleate closely resemble those observed in plasma, which in turn directly reflect adipose fatty acids [30]. This suggests that palmitoleate liberated from adipose tissue, is the underlying mechanism for 16∶1n7 enrichment in skeletal muscle in FABP^−/−^ mice. In agreement with similar regulation of SCDs in WAT and muscle, are the previously observed decreased expression levels of SCD1 and 2 in muscle of HSL null mice [32].

Studies on FABP^−/−^ mice have suggested that the majority of palmitoleate in liver cannot be accounted for by adipose-derived free fatty acids and that circulating 16∶1n7 may instead play a regulatory role on the lipogenic program of the liver rather than serving as a substrate to drive TAG synthesis [30]. It has further been demonstrated that suppression of SCD1 activity is a key factor determining the liver steatosis phenotype in FABP^−/−^ mice. In the HSL null mice, the opposite scenario of significantly decreased proportions of palmitoleate in WAT and plasma prevent a suppression of SCD1 in the liver, resulting in increased expression levels of liver SCD1. A consequence of this is elevated proportions of 18∶1n9 [23] and increased TAG incorporation resulting in liver steatosis [5], a possible contributing factor to the decreased insulin sensitivity observed in HSL null mice on HFD (**Figure 8**)**.** Decreased levels of SCD1 and SCD2 in WAT lead to lower amount of palmitoleate. An absence of lipid chaperons has been shown to positively regulate de novo lipogenesis in WAT, disconnecting the normal response of this tissue to a HFD [30]. Although decreased levels of aP2 (FABP4), the major lipid chaperon in WAT, is seen in HSL null mice fed a HFD [4], lipogenesis is decreased. It is possible that a total absence of lipid chaperons is necessary to get an effect on lipogenesis and that the remaining aP2 and/or other lipid chaperones in WAT are still able to mediate the negative effect of HFD on SCD1 levels. Also, severely decreased levels of other transcription factors known to positively regulate SCD1 expression, e.g. PPARγ and SREBP1c, in WAT of HSL null mice (**Figure 7A**) [4], [5] likely influence the amount of SCD1 in adipose tissue of HSL null mice.

In WAT from HSL deficient mice, a large increase in the expression of Elovl1 is seen, which is surprising as this isoform is previously described to be constitutively expressed [7]. Consistent with this is an increased elongation of 16∶0 to 18∶0 in WAT and plasma from HSL null mice. Proportions of 16∶0 are not increased in WAT from HSL null mice (**Table 2**) despite markedly decreased levels of desaturases. Decreased de novo lipogenesis in WAT, suggested by dramatically decreased protein levels of SREBP1 and FAS (**Figure 7**), could counteract the accumulation of 16∶0. Also, increased elongation of 16∶0 by Elovl1 in WAT could help prevent a buildup of 16∶0 in favor of 18∶0, the latter of which is significantly increased in WAT (1.23, p<0.01) (**Table 2**).

Similar to WAT, there is an increased ratio of 18∶0/16∶0 in the liver of mice deficient in HSL. Surprisingly, the mRNA levels of Elovl6 were unchanged between the two genotypes (**Table 1**). However, although Elovl6 is shown to account for a majority of 16∶0 elongation in the liver [25], the increased expression of Elovl1 and 3 seen in the liver of HSL null mice might influence the elongation of 16∶0.

Many models of mice with protection against diet-induced insulin resistance also display resistance to diet-induced obesity. However, a few studies showing maintained insulin sensitivity despite diet-induced obesity and hepatosteatosis exist, e.g. FABP4 and 5 [33], [34] as well as Elovl6 [25] deficient mice. The uncoupling of obesity from insulin resistance, highlights the importance of tissue fatty acid composition for insulin sensitivity and further suggests that conversion of palmitate to stearate, and the desaturation of stearate to oleate are critical events for the emergence of insulin resistance. HSL deficient mice are protected against HFD-induced adiposity but not liver steatosis and insulin resistance. This model supports the role of SCD1 and the level of desaturation as an important metabolic controller, regulating the amount of lipid being stored in various tissues. In WAT, an absence of HSL, via decreased levels of desaturases, suppresses the incorporation of FAs into TAG, decreasing lipid storage. Meanwhile, in the liver, where increased desaturation occurs, the opposite scenario is seen.

The exact mechanism of how an absence of HSL will affect the expression of key enzymes involved in lipid metabolism, with profound effects on the lipid profile, is still unknown. We have previously suggested that HSL appears to play an important role in the generation of lipids for transcriptional regulation and various lipid signaling events [6]. Recently, it has also been postulated that HSL modulate adipose lipid metabolism by providing intrinsic ligands or pro-ligands for PPARγ [35]. Selective mobilization of fatty acids by HSL according to chain length and degree of desaturation has also been demonstrated [36]. As previously speculated in the context of cholesterol metabolism [23], a cross-talk exists between adipose tissue and the liver, more pronounced in the fasted state when HSL is maximally active. By inactivating the gene for HSL, the communication between WAT and the liver is disturbed, greatly affecting lipid metabolism in these tissues reflected in the severely altered plasma lipid profile (**Figure 6A**). This study supports the role of 16∶1n7 as a possible mediator of this crosstalk between adipose tissue and liver, and that dysregulation of this mediator will have profound effects on lipid metabolism and insulin sensitivity.

In conclusion, this study illustrates the importance of HSL for normal lipid metabolism in response to a HFD. An absence of HSL has a great impact on the expression and activity of elongases and desaturases, resulting in altered lipid profiles in WAT, liver and plasma. Finally, altered levels of the recently suggested lipokine palmitoleate in tissue and plasma of HSL null mice, could be an important factor mediating and contributing to the changed lipid profile, and possibly also to the decreased insulin sensitivity seen in HSL null mice on a HFD. Further investigations into the role of HSL for the generation of lipids involved in crucial metabolic processes is warranted in order to better understand and combat the complex processes of obesity and insulin resistance.
